# A Sensitive Reporter Mouse Model to Study Adipocyte‐Derived Extracellular Vesicles In Vivo

**DOI:** 10.1002/jev2.70243

**Published:** 2026-02-19

**Authors:** Didde Riisager Hansen, Rugivan Sabaratnam, Lasse Bach Steffensen, Per Svenningsen

**Affiliations:** ^1^ Department of Molecular Medicine University of Southern Denmark Odense Denmark; ^2^ Steno Diabetes Center Odense Odense University Hospital Odense Denmark; ^3^ Department of Clinical Research University of Southern Denmark Odense Denmark

**Keywords:** adipose tissue, CD9, CD63, Cre recombinase, EV reporter, nanoluciferase, obesity

## Abstract

Extracellular vesicles (EVs) affect the function of cells in living animals. Yet, cell type‐specific EV abundances and their distribution in biological fluids are technically challenging to study. Thus, we aimed to develop an in vivo EV reporter system to monitor cell‐type‐specific EVs, with a focus on adipocyte‐derived EVs. While our previously generated EV reporter construct had insufficient sensitivity, we successfully created a sensitive EV reporter using an adeno‐associated virus (AAV) vector with Cre‐activated expression of human CD63 fused to NanoLuc (CD63‐NanoLuc). Moreover, we designed a control AAV construct for monitoring constitutive secretion of NanoLuc (sec‐NanoLuc). AAV administration to mice induced adipocyte‐specific expression of both reporters. NanoLuc activity was detected in plasma. While sec‐NanoLuc was predominantly in plasma and urine, CD63‐NanoLuc was highest in adipose tissues (ATs). We challenged mice with a 2‐week high‐fat diet (HFD), which had minimal effects on body weight and adipogenic markers. Still, the HFD‐fed CD63‐NanoLuc mice, but not sec‐NanoLuc mice, showed significantly higher NanoLuc activity in ATs, lungs, kidneys and urine. Thus, our CD63‐NanoLuc and sec‐NanoLuc constructs revealed an early effect of HFD on the abundance and distribution of adipocyte‐derived EVs and provide a sensitive system for monitoring cell‐type‐specific EVs in health and disease.

## 1 Introduction

Extracellular vesicles (EVs) containing RNAs and proteins from their parental cell are secreted directly to the extracellular fluids, such as interstitial fluid and blood plasma (Auber and Svenningsen 2022; Li et al. 2022). The body fluids, therefore, contain a highly mixed population of EVs derived from diverse cell types, and a significant challenge is to untangle the cell type‐specific EV abundances to better understand the physiological role of EVs. Yet, the level of cell type‐specific EVs is often low (Hansen and Svenningsen 2024) and affected by multiple factors, such as cell type abundance and the cell type's EV secretion rate (Auber and Svenningsen 2022). Moreover, anatomical restrictions also affect the biodistribution of EVs. We have, for example, demonstrated that in EV reporter mice, cardiomyocyte‐derived EVs are readily detectable in plasma but not urine samples. In contrast, the opposite is true for kidney epithelial‐derived EVs (Norgard et al. 2022). Thus, monitoring changes in cell type‐specific EV abundances and their biodistribution in, for example, disease models is challenging, and the lack of these details limits our knowledge of the physiological role of EVs and their biodistribution in health and disease.

EVs derived from adipocytes have been intensively studied and may play a significant role in the pathogenesis of obesity‐associated disorders. In obesity, adipose tissue (AT)‐derived EVs are enriched in proteins and miRNAs associated with increased glucose intolerance, insulin resistance and inflammation, including polarization of pro‐inflammatory M1 macrophages (Kulaj et al. 2023; Kita et al. 2019; Yang et al. 2023; Pan et al. 2019; Kwan et al. 2021), and the effects linked to obese AT‐derived EV‐associated miRNAs were reversed by specific miRNA knockdown in mice (Kulaj et al. 2023; Yang et al. 2023; Pan et al. 2019; Zarkesh et al. 2022; Liu et al. 2019). EVs enable the transfer of adipocyte‐derived nucleic acids and proteins to local and distant tissues (Han et al. 2024). Locally in ATs, EVs mediate a regulated transfer of cellular material between endothelial cells and adipocytes (Crewe et al. 2018) and between resident macrophages and adipocytes (Liu et al. 2019; Ying et al. 2017; Gao et al. 2021). Adipocyte‐derived EVs can also affect more distant tissues. Mice intravenously injected with donor AT‐derived EVs from obese mice or diabetic humans showed significantly impaired cognitive function compared to healthy donor EVs (Wang et al. 2022). Similarly, endogenous adipocyte‐derived EVs transferred oxidatively damaged but functional mitochondrial particles to cardiomyocytes (Crewe et al. 2021). The EV‐encapsulated mitochondrial particles induce oxidative stress within the cardiac tissue, producing an initial antioxidant response with cardiomyocyte protective effects. Still, long‐term exposure to EV‐induced oxidative stress leads to cardiac damage (Crewe et al. 2021). Thus, not only the molecular cargo transferred by adipocyte‐derived EVs but also how, for example, adipocyte‐derived EV abundance and their biodistribution changes during obesity, are essential to understand.

White adipocytes in culture secrete EVs at a relatively high rate (Garcia‐Martin et al. 2022), and obesity can induce oxidative stress in adipocytes, further stimulating EV release (Auber and Svenningsen 2022; Norgard et al. 2023; Sabaratnam et al. 2023). Indeed, chemogenetic induction of reactive oxygen species (ROS) production in adipocytes increased serum EV levels in mice (Crewe et al. 2021), and obesity in humans and mice is associated with higher circulating plasma EV levels (Kwan et al. 2021; Crewe et al. 2021; Eguchi et al. 2016). Although the higher EV concentration in obese individuals correlated with glucose intolerance and insulin resistance (Eguchi et al. 2016), the higher plasma EV abundance in obesity is unlikely to rely only on increased adipocyte EV secretion because their plasma levels are <1% of the total circulating EV pool (Auber and Svenningsen 2022; Li et al. 2020). Thus, to determine how adipocyte‐derived EV abundances and their biodistribution change during obesity, we need new experimental methods to track and quantify circulating cell‐type specific EVs in a living organism.

Current in vivo EV reporter systems for monitoring cell type‐specific EV abundance generally consist of a Cre recombinase‐activated construct expressing a tetraspanin, for example, CD9, CD63, or CD81, fused to a reporter protein (Norgard et al. 2022; Estrada et al. 2022; Li et al. 2022; Welsh et al. 2024). The reporter protein is often a fluorescent protein such as enhanced green fluorescent protein (EGFP), which enables the tracking of cell‐to‐cell transfer of EVs and monitoring plasma EV levels of abundant EVs (Norgard et al. 2022; Chen et al. 2024). Yet, the abundance of cell type‐specific EVs is low and can, moreover, be restricted by intercompartmental barriers. This renders the fluorescent‐based reporter systems' low sensitivity and challenges the knowledge translation from in vitro studies to an in vivo setting. In this study, we aim to develop an EV reporter system for monitoring plasma adipocyte‐derived EV abundance and biodistribution during the early phase of high‐fat diet (HFD)‐induced obesity. We initially used the CD9‐EGFP reporter mouse; however, the sensitivity was too low. We, therefore, developed a new EV reporter based on the bioluminescent protein Nanoluciferase (NanoLuc) with high sensitivity for in vivo tracking (Gupta et al. 2020; Luo et al. 2020; Boudna et al. 2024). The design of the EV reporter also allowed us to include an additional control recommended by the MISEV2023 guidelines (Welsh et al. 2024), which enables the normalization of EV abundance in biofluids (Royo et al. 2020; Vaiaki and Falasca 2024) and monitoring the cell type‐specific EV abundance and distribution changes compared to a secreted protein. We used this sensitive EV reporter system to investigate the abundance and biodistribution of adipocyte‐derived EVs in plasma and urine following 2 weeks of HFD.

## 2 Materials and Methods

### 2.1 EV Track Data

We have submitted all relevant data to the EV‐TRACK platform (EV‐TRACK ID: EV250023).

### 2.2 Constructs and Plasmids

The already established EV reporter protein CD9‐EGFP was previously generated by fusing mouse CD9—truncated after the first 117 amino acids (ending at the third transmembrane domain)—to EGFP (Norgard et al. 2022). We designed the EV reporter (CD63‐NanoLuc) and a secreted reporter protein (sec‐NanoLuc) by genetically fusing human CD63 or IgGκ signal peptide to Nanoluciferase (NanoLuc) and hemagglutinin (HA), driven by a constitutively active EF1A or EFS promoters, respectively. The transgenes (*pEF1A‐CD63‐NanoLuc‐HA* or *pEFS‐IgGκ‐NanoLuc‐HA*) were inserted into a PiggyBac backbone with a CMV‐driven EGFP‐Puromycin gene. For in vivo applications, the *CD63‐NanoLuc‐HA* and *IgGκ‐NanoLuc‐HA* transgenes were inserted with a double‐floxed inverted orientation in an adeno‐associated vector (AAV), ensuring transgene activation only in Cre recombinase‐expressing cells. We also designed an AAV plasmid with Cre recombinase driven by a short human adiponectin promoter (hAdipoq‐Cre) (Shamsi et al. 2020) using a chicken β‐actin (CBA)‐WPRE backbone. All vectors were synthesized by VectorBuilder. The mRFP‐Rab5 was a gift from Ari Helenius (Addgene, 14437), and mCherry‐ER (Addgene, 55041) and mTagBFP2‐CD81‐10 (Addgene, 55281) were gifts from Michael Davidson.

### 2.3 Cell Cultures

HEK293T cells were transiently transfected with CD63‐NanoLuc, sec‐NanoLuc, mRFP‐Rab5, mCherry‐ER or mTagBFP2‐CD81‐10 using Metafectene Pro (Biontex, T040). Cells were maintained in Dulbecco's Modified Eagle Medium, F‐12 (DMEM‐F12, Gibco) with 10% fetal bovine serum (FBS, Fisher Scientific) and 1% Penicillin‐Streptomycin in a 5% CO_2_ humidified incubator at 37°C. EGFP fluorescence was used to verify successful transfection. Cells were harvested in 1X RIPA Lysis Buffer (Millipore, 20‐188) with Halt Protease and Phosphatase Inhibitor Cocktail (Thermo Scientific, 78441), incubated on a shaker for 60 min at 4°C and centrifuged at 13,000 × *g* for 10 min at 4°C. The cell‐conditioned medium was added Protease Inhibitor Cocktail (Sigma‐Aldrich, P8340) and harvested by centrifugation at 5000 × *g* for 10 min at 4°C. Samples were stored at −80°C.

### 2.4 AAV Production

The protocol for AAV production in HEK293T cells was adapted from Addgene. Following AAVs were produced: rAAV9‐hAdipoq‐Cre, rAAV9‐CAG‐CD63‐NanoLuc‐HA and rAAV9‐EFS‐IgGκ‐NanoLuc‐HA. Cells were grown to 80% confluency in 15 cm petri dishes in a 5% CO_2_ humidified incubator at 37°C. HEK293T cells were triple transfected with plasmid of interest (100 µg), RepCap (100 µg) and pHelper (200 µg) plasmid in a PBS‐PEI mix (0.0323 mg/mL, pH 2, Polyethylenimine, Sigma‐Aldrich, 408727). Two days post transfection, cells and conditioned medium were harvested by trypsination. Samples were centrifuged at 4800 × *g* for 10 min at 4°C. The supernatants were PEG precipitated (0.4 g/mL PEG, PEG‐8000, Sigma‐Aldrich, 89510, 0.146 g NaCl/mL) overnight at 4°C, centrifuged at 4800 × *g* for 10 min at 4°C and resuspended in 0.001% Pluronic F68 (Gibco, 1150616, 1X PBS, 200 mM NaCl). Cells were lysed by three freeze‐thaw cycles and cleared cell lysates were treated with Benzonase (Sigma‐Aldrich, E8263) to digest genomic DNA. AAV purification was done by Iodixanol gradient ultracentrifugation (350,000 × *g*, 2 h and 25 min, 18°C). Purified AAVs were treated with DNAse I (Sigma‐Aldrich, 4716728001) for 1 h at 37°C to degrade non‐viral DNA. Viral DNA was released by Proteinase K (Sigma‐Aldrich, 03115828001) digestion for 2 h at 50°C and 500 rpm followed by Proteinase K inactivation at 95°C for 10 min at 500 rpm. AAV concentrations were quantified according to Addgene's protocol for AAV titration qPCR using 5x HOT FIREPol EvaGreen qPCR Mix Plus (Thistle Scientific, 08‐24‐00001‐5) following manufacturer's protocol. A dilution standard was made using an internal standard with a known AAV concentration. Primers used for quantification were: *AAV2 ITR*: 5′‐GGAACCCCTAGTGATGGAGTT and 5′‐CGGCCTCAGTGAGCGA. AAVs were stored at −80°C.

### 2.5 Animal Studies

To test if AAVs could be used for adipocyte‐specific EV labelling with CD9‐EGP, we generated the rAAV9‐hAdipoq‐Cre/CD9truc‐EGFP model by a single intraperitoneally (IP) injection of rAAV9‐hAdipoq‐Cre into 6‐weeks‐old CD9truc‐EGFP^wt/tg^ (C57BL/6J) mice (Norgard et al. 2022). We generated the Adipoq‐Cre × CD9truc‐EGFP model by crossbreeding CD9truc‐EGFP^wt/tg^ mice with Adipoq‐Cre^tg^ mice (C57BL/6J; FVB/NJ, The Jackson Laboratory, 010803). Primers used for genotyping were: *CD9truc‐EGFP* locus: 5′‐TGCACCTCAAACACTCAAGC and 5′‐TCTTCCTGGAACACAGCTCA; *Adipoq‐Cre* locus: 5′‐GGATGTGCCATGTGAGTCTG and 5′‐ACGGACAGAAGCATTTTCCA. We created the CD63‐NanoLuc and sec‐NanoLuc mouse models by a single IP injection of rAAV9‐CAG‐CD63‐NanoLuc‐HA or rAAV9‐EFS‐IgGκ‐NanoLuc‐HA into 6‐weeks‐old Adipoq‐Cre^tg^ mice. For all studies, the mice were housed in a 12:12 h light/dark cycle with balanced temperature (21 ± 2°C) and humidity (55 ± 2%), with free access to tap water and a standard rodent diet (Lab Diet 5001, Lab Supply). Two to three weeks post‐injection of the CD63‐NanoLuc and sec‐NanoLuc AAVs, the mice were put on a chow diet (Lab Diet 5001, Lab Supply) or HFD (D12492, 60% kcal from fat, Research Diets) ad libitum for 2 weeks. Genders were equally distributed between diet groups. All animal studies were performed in accordance with Danish Law and approved by the Danish Animal Experimentation Council (2021‐15‐0201‐01066) following the ARRIVE guidelines.

### 2.6 Sample Collection

Spot urine was collected, and mice were anesthetized by IP injection with 10 mg/kg Xylazine and 50 mg/kg Ketamine. Blood was harvested in EDTA vials (Sarstedt, Germany) by cardiac puncture, and plasma was extracted by centrifugation at 4000 × *g* for 10 min at 4°C. Mice were flushed with sterile PBS (1X, Gibco, 14200067) through the left ventricle. For immunohistochemistry, tissues were immersed in 4% formaldehyde for 24 h and stored in PBS (1X, Gibco) with 0.05% sodium azide at 4°C until paraffin embedment. For proteomics and transcriptomics, tissues were snap‐frozen in liquid nitrogen. Tissue was homogenized in 1x RIPA Lysis Buffer supplemented with Halt Protease and Phosphatase Inhibitor Cocktail (Thermo Scientific). Samples were kept on ice for 1 h and centrifuged at 12,000 × *g* for 10 min at 4°C. Protein concentration was determined using Pierce BCA Protein Assay.

### 2.7 EV Isolation From Plasma and Medium

We collected fractions 1–20 of cell‐conditioned medium and fraction 6–10 of plasma using a qEV original 35 nm GEN 2 column (Izon Science, ICO‐35). Fractions were stored at −80°C. Plasma and conditioned medium were added ExtraPEG (Rider et al. 2016) (16% PEG‐6000, Sigma Aldrich, 1 M NaCl, Milli‐Q water) and incubated rotating overnight at 4°C. Samples were centrifugated at 5000 × *g* for 15 min at 4°C. Plasma was immunopurified using GFP‐Trap Magnetic Particles M‐270 (ChromoTek GmbH). The pellet was resuspended in 1x RIPA Lysis Buffer for Western Blotting.

### 2.8 Immunoblotting

Samples were added 4X LDS Sample Buffer and 10X Sample Reducing Agent (NuPAGE, Invitrogen, Thermo Fischer Scientific) and heated at 95°C for 5–10 min. Separated proteins were transferred to a 99% ethanol‐activated PVDF membrane. The membrane was blocked in 5% skim milk in TBS‐T (20 mM Tris‐base, 137 mM NaCl, 0.05% Tween‐20 (Merck), pH 7.6) for 30 min and incubated with primary antibody (Table 1) overnight at 4°C. The membrane was washed in TBS‐T and incubated with a secondary antibody (Table 1) for 1 h at room temperature. Proteins were stained with Ponceau S Staining Solution (Thermo Scientific, A40000279) for 10 min and washed in Milli‐Q at room temperature. Proteins were visualized using Western Lightning ECL Pro (Perkin Elmer) and the iBright CL1500 Imaging System (Invitrogen) for data analysis and quantification. The cellular ALIX and Flotillin‐1 protein expression within conditioned medium was normalized to the cellular protein concentration.

**TABLE 1:** Primary and secondary antibodies for Western blotting, immunocytochemistry and immunohistochemistry.

Target protein	Origin	Company	Catalog no.	Dilution
Western Blot				
Primary antibodies				
GFP	Rabbit	GeneTex	GTX113617	1:1000
HA‐tag (C29F4)	Rabbit	Cell Signaling	3724	1:1000
Human CD63	Mouse	DSHB	H5C6	1:1000
Alix (3A9)	Mouse	Cell Signaling	2171	1:2000
Flotillin‐1	Goat	Abcam	Ab13493	1:1000
Secondary antibodies				
Goat anti‐mouse immunoglobin/HRP		DAKO	P0447	1:2000
Goat anti‐rabbit immunoglobin/HRP		DAKO	P0448	1:2000
Rabbit anti‐goat immunoglobin/HRP		DAKO	P0449	1:2000
Immunocytochemistry and immunohistochemistry				
Primary antibodies				
GFP	Chicken	Abcam	Ab13970	1:1000
HA‐tag (C29F4)	Rabbit	Cell Signaling	3724	1:200
Human CD63	Mouse	DSHB	H5C6	1:500
F4/80	Rabbit	Abcam	Ab111101	1:250
Secondary antibodies				
Anti‐chicken IgY HRP		Promega	G135A	1:200
Goat anti‐rabbit immunoglobin/HRP		DAKO	P0448	1:200
Goat anti‐mouse immunoglobin/HRP		DAKO	P0447	1:200
Goat anti‐mouse IgG (H+L), Alexa Fluor 647		Invitrogen	A21235	1:200
Goat anti‐rabbit IgG (H+L), Alexa Fluor 488		Invitrogen	A11034	1:200

### 2.9 Immunocytochemistry

Cells were seeded on coverslips in a 12‐well plate (Biocoat Cell environments, Poly‐d‐Lysine Cellware) and transfected with plasmid. Next, cells were fixed in 4% formaldehyde for 10 min and rinsed in PBS (1X, Gibco) with 1 mM MgCl_2_ and 0.1 mM CaCl_2_. Cells were permeabilized by 0.5% Triton X‐100 (Sigma‐Aldrich, 1X PBS, Gibco) for 10 min followed by rinsing cells in PBS (1X, Gibco). Cells were blocked in 1% Bovine Serum Albumin (BSA, VWR, 1X PBS, Gibco) for 1 h followed incubation with primary antibody (Table 1) in 1% BSA (VWR, 1X PBS, Gibco) overnight at 4°C. Slides were washed in PBS (1X, Gibco) and incubated with secondary antibody (Table 1) for 1 h at room temperature. Slides were washed in PBS (1X, Gibco) and incubated with DAPI (Sigma‐Aldrich, D9542, 1:10,000 in PBS) for 5 min, washed in PBS (1X, Gibco), and mounted with Dako Fluorescence Mounting Medium (DAKO, S3023). Imaging was done on the Nikon Ti2 Widefield microscope using the NIS‐Elements software (with JOBS module).

### 2.10 Immunohistochemistry

Sectioned tissue (5 µm) was deparaffinated in xylene, rehydrated in ethanol (99%–70%), and boiled in TEG buffer (1 mmol/L Tris, 0.5 mM EGTA, pH 9.0) for 15 min. After 20 min on ice, endogenous peroxidase was blocked by 0.3% H_2_O_2_ (1X PBS, Gibco, 50 mM NH_4_Cl) for 10 min. Slides were washed in PBS (1X, Gibco), blocked in 0.3% Triton X‐100 (Sigma‐Aldrich, 1X PBS, Gibco) for 30 min, and incubated with primary antibody (Table 1) in 0.3% Triton X‐100 overnight at 4°C. Slides were washed in PBS‐T (1X PBS, Gibco, 0.05% Tween‐20 (Merck)) and incubated with a secondary antibody (Table 1) in PBS‐T for 1 h at room temperature. Slides were washed in PBS‐T (1X PBS, 0.05% Tween‐20) and PBS (1X, Gibco), stained in DAB (3,3′‐diaminobenzidine) and haematoxylin, and mounted with Aquatex (Merck kGaA, Darmstadt, Germany).

For immunofluorescence double staining, slides pre‐incubated with primary and secondary antibody (Table 1) were incubated with TSA Vivid Fluorophore Kit 570 (Tocris, 7526). Slides were then boiled in TEG buffer (1 mmol/L Tris, 0.5 mM EGTA, pH 9.0) for 6 min, cooled on ice for 20 min, washed in PBS (1X, Gibco), incubated with new primary antibody (Table 1), washed in PBS‐T, and incubated with secondary antibody (Table 1) and washed in PBS‐T. Afterwards, slides were incubated with DAPI (Sigma‐Aldrich, D9542, 1:10,000 in PBS‐T) for 5 min, washed in PBS (1X, Gibco), and mounted with Dako Fluorescence Mounting Medium (DAKO, S3023). Imaging was done using the Olympus BX51 microscope.

### 2.11 Quantitative Real‐Time PCR

RNA was extracted using TRIzol Reagent (Invitrogen, 15596026). For easier detection of viral DNA, RNA was treated with RNase A (Thermo Scientific, R1253) at 37°C for 2 h in a Thermal Cycler. To quantify mRNA expression of adipocyte markers, RNA was treated with DNase I (Thermo Scientific, EN0521) followed by cDNA synthesis using iScript cDNA Synthesis Kit (BioRad, 1708890). Quantitative PCR was performed using iTaq Universal SYBR Green Supermix (BioRad, 1725124). Using the AriaMX Real‐Time PCR system (VWR), we detected viral DNA in RNase‐treated samples using following primers: *Cre‐induced flip of construct*: 5′‐TTACGCTTAAGGTGGCCCTA and 5′‐CACATAGCGTAAAAGGAGC; *Internal control for plasmid DNA*: 5′‐AGAATGCGTTCGCACAGCC and 5′‐GGCCGTATGAAGGCATCGCC. The mean 2^−Ct^ value was normalized to the mean 2^−Ct^ of reference wild‐type tissue (*n* = 4–5). We used the QuantStudio 3 Real‐Time PCR system (Thermo Scientific) to quantify gene expression of adipogenic markers in cDNA using the primers *Adipoq*: 5′‐ACTTGTGCAGGTTGGATGGC and 5′‐CCCTTCAGCTCCTGTCATTCC; *Fabp4*: 5′‐AGGCCTGGCCTTTGACTTAGA and 5′‐TGAGGCAGTTTGACCATTTTATTCT; *Pparg2*: 5′‐ CTCTGTTTTATGCTGTTATGGGTGA and 5′‐GGTCAACAGGAGAATCTCCCAG; *Sfrs4* (reference gene): 5′‐AGCCGGAGTGGTAGCAGTAA and 5′‐ACTGCGGCTCTTGTTGTCTT. We normalized the mean 2^−Ct^ values to the reference gene and calculated the fold change to the chow group.

### 2.12 EV Analyses

The cell‐conditioned medium was treated with 10% Triton X‐100 (Sigma‐Aldrich, T9284) or Milli‐Q for 30 min at room temperature. Samples were treated with Proteinase K (Invitrogen, AM2542) at 37°C for 30 min. Luciferase activity in tissues, body fluids, and cell‐conditioned medium was measured using the Nano‐Glo Luciferase Assay System (Promega, N1110). Luminescence was measured in Relative Light Units per second (RLU/s) using the Orion L Microplate luminometer and Simplicity 4.10 software. The luminescence of tissues, cells and medium was normalized to the cellular protein concentrations. No normalization was done for plasma and urine samples.

### 2.13 Statistics and Graphs

A Shapiro–Wilk test was used to test for normality. Parametric tests included two‐sample and paired *t*‐tests, and repeated measures ANOVA followed by pairwise *t*‐tests. Data is represented as mean ± standard deviation (SD). Non‐parametric tests included Mann–Whitney *U* tests. Data is represented as median and interquartile range (IQR). We adjusted for multiple tests using Benjamini–Hochberg correction. All statistical analyses and graphs were made in R Studio using R version 4.4.2 (2024). Significance levels are indicated with ns (non‐significant), * (*p* < 0.05), ** (*p* < 0.01) and *** (*p* < 0.001). Figures were made in Adobe Illustrator with templates from SVG Repo, 2025—CC BY 4.0.

## 3 Results

### 3.1 CD9‐EGFP Is Insufficient to Detect Adipocyte‐Derived Plasma EVs

**FIGURE 1:**
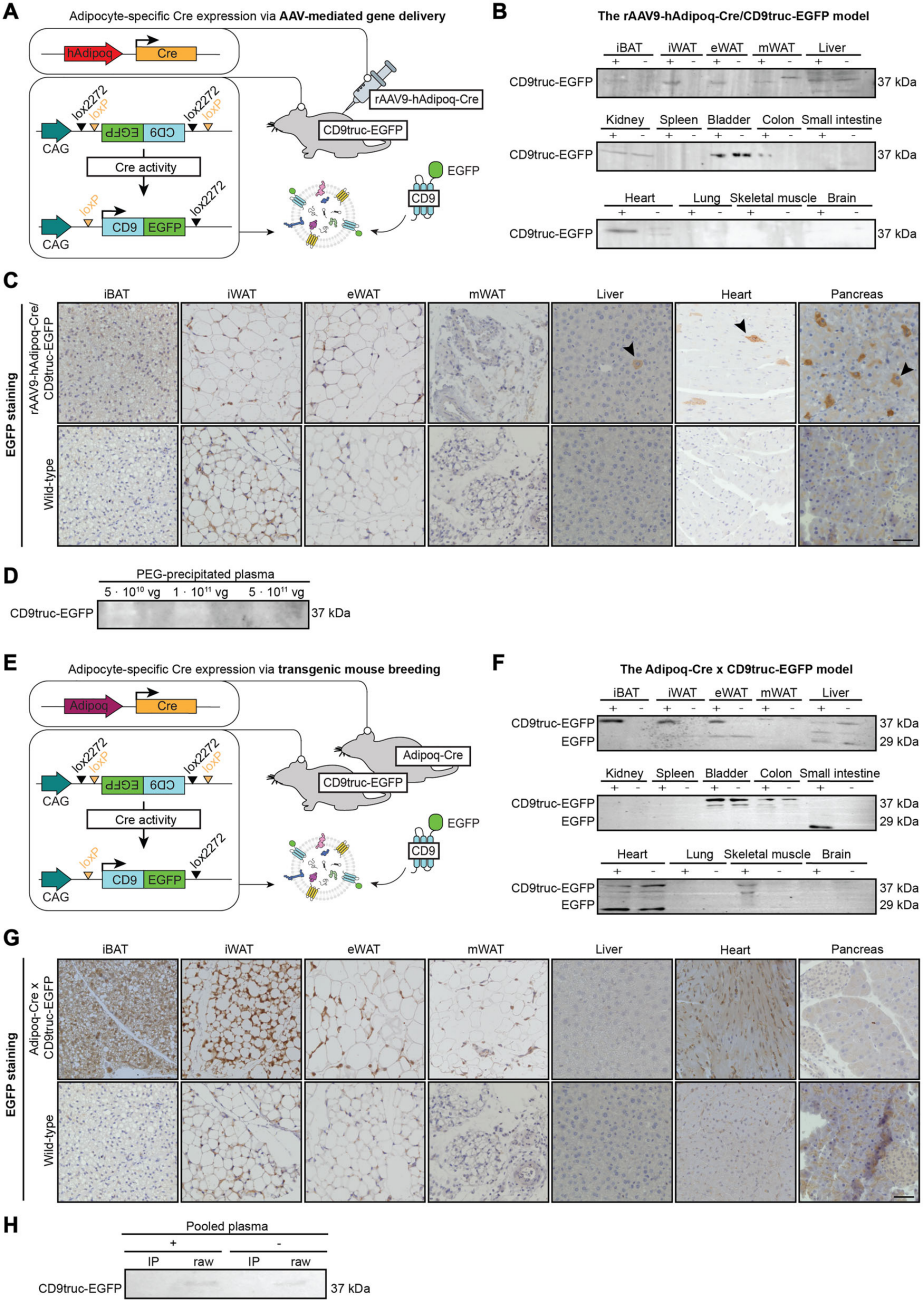
The CD9‐EGFP‐based EV reporter system for labelling adipocyte‐derived EVs. (A) Adipocyte‐specific Cre expression in CD9truc‐EGFP reporter mice was achieved via AAV‐mediated gene delivery of Cre recombinase driven by a short human adiponectin (hAdipoq) promoter. In the presence of Cre, the double‐floxed oriented CD9truc‐EGFP transgene is recombined and activated, leading to the expression of EGFP‐labelled CD9‐positive EVs driven by a constitutively active CAG promoter. (B) Protein expression of CD9truc‐EGFP in rAAV9‐hAdipoq‐Cre/CD9truc‐EGFP mice (+) was detected in adipose tissues, that is, interscapular brown adipose tissue (iBAT), inguinal white adipose tissue (iWAT) and epididymal white adipose tissue (eWAT), and in the heart, compared to wild‐type tissues (−). Full blots are shown in Figure S6A–C. (C) EGFP staining of tissues from rAAV9‐hAdipoq‐Cre/CD9truc‐EGFP mice showed vague signals in adipose tissues and off‐target effects in the liver, heart and pancreas compared to wild‐type tissues. The scalebar represents 50 µm. (D) CD9truc‐EGFP protein expression was not detected in PEG‐precipitated plasma samples from chow‐fed rAAV9‐hAdipoq‐Cre/CD9truc‐EGFP mice receiving different doses of AAVs, that is, 5 × 10^10^ viral genomes (vg), 1 × 10^11^ vg and 5 × 10^11^ vg. The full blot is shown in Figure S6D. (E) To rule out that undetectable CD9truc‐EGFP expression in plasma was due to low adipocyte transduction with AAV9, the CD9truc‐EGFP mice were crossbred with Adipoq‐Cre mice. (F) CD9truc‐EGFP protein expression was detected in adipose tissues, that is, iBAT, iWAT and eWAT of Adipoq‐Cre × CD9truc‐EGFP mice (+), compared to wild‐type tissues (−). CD9truc‐EGFP and free EGFP were also detected in skeletal muscle tissue and small intestines. Full blots are shown in Figure S6E–G. (G) Protein expression of CD9truc‐EGFP was stronger in adipose tissues from Adipoq‐Cre × CD9truc‐EGFP mice than for rAAV9‐hAdipoq‐Cre/CD9truc‐EGFP mice. The scalebar represents 50 µm. (H) Despite strong adipose tissue expression in Adipoq‐Cre x CD9truc‐EGFP mice, CD9truc‐EGFP was not detected in raw plasma or by immunoprecipitation (IP) of pooled plasma from seven mice. The full blot is shown in Figure S6H.

We have previously successfully used a CD9‐EGFP based EV reporter system to monitor kidney epithelium‐ and cardiomyocyte‐derived EV abundance in urine and plasma samples by crossbreeding with kidney‐ and cardiomyocyte‐specific Cre‐driver strains (Norgard et al. 2022; Chen et al. 2024). To circumvent time‐consuming crossbreeding, we tested whether AAV‐mediated Cre delivery could be used to induce adipocyte‐specific labelling of EVs with CD9‐EGFP (Figure 1A). We, therefore, designed an AAV‐applicable construct with Cre driven by a truncated version of the human adiponectin promoter (hAdipoq) (Shamsi et al. 2020). IP injection of rAAV9‐hAdipoq‐Cre at a dose of 5 × 10^11^ vg in mice induced CD9‐EGFP expression in different AT depots but not in the liver (Figure 1B and Figure S1A). This dose showed specific CD9‐EGFP protein expression in interscapular brown AT (iBAT), inguinal white AT (iWAT), and epididymal white AT (eWAT) (Figure 1B). The liver from AAV‐mediated CD9‐EGFP mice had few EGFP‐positive cells (Figure 1C and Figure S1B), and EGFP immunoreactivity was also observed in the heart and pancreas, which do not express adiponectin and, therefore, likely represent leaky expression (Figure 1B , C and Figure S1B). Despite tissue expression, CD9‐EGFP was below the detection limit in PEG‐precipitated plasma samples from chow‐fed mice receiving different doses of AAV (Figure 1D), even after GFP‐immunoprecipitation of plasma from chow‐fed mice (*n* = 7) (Figure S1C). Similarly, CD9‐EGFP was not detected in plasma from chow‐ or HFD‐fed mice (*n* = 3) (Figure S1D), or in plasma from chow‐fed mice receiving a dose of 1 × 10^12^ vg of rAAV9‐hAdipoq‐Cre (Figure S1E). To rule out that the lack of CD9‐EGFP detection in plasma samples is due to low adipocyte transduction with AAV9, we crossbred CD9‐EGFP mice with Adipoq‐Cre mice (Figure 1E). CD9‐EGFP protein expression was restricted to ATs (Figure 1F,G and Figure S1F); however, the adipocyte‐derived EVs were still undetectable in pooled and GFP‐immunoprecipitated plasma samples (*n* = 7) (Figure 1H), indicating that the sensitivity of the CD9‐EGFP reporter system was too low to enable monitoring of circulating adipocyte‐derived EVs.

### 3.2 Validation of NanoLuc and HA‐Tag as EV‐Encapsulated Cargo

**FIGURE 2:**
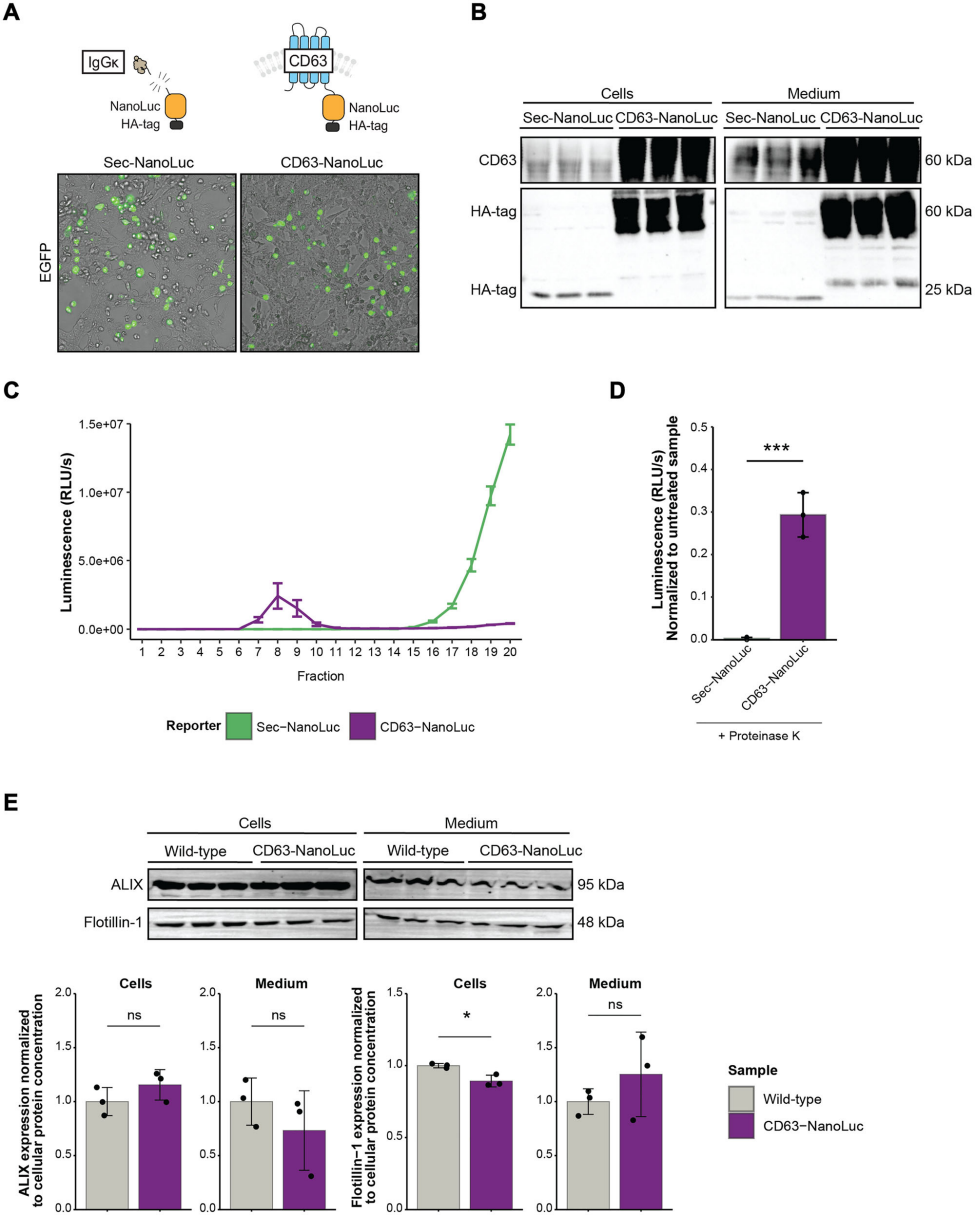
In vitro characterization of the CD63‐NanoLuc and sec‐NanoLuc system in transiently transfected HEK293 cells. (A) Fluorescence microscopy of EGFP confirmed successful transfection with CD63‐NanoLuc and sec‐NanoLuc constructs. (B) Human CD63 and HA‐tag protein expression was detected in cells and cell‐conditioned medium from CD63‐NanoLuc and sec‐NanoLuc expressing cells. Full blots are shown in Figure S6I–J. (C) Luciferase activity (RLU/s) was detected in fractions 8–10 (containing EVs) and fraction > 16 (containing proteins) of CD63‐NanoLuc (purple, *n* = 3) and sec‐NanoLuc (green, *n* = 3) cell‐conditioned medium separated by size‐exclusion chromatography, respectively. Error bars represent mean ± SD (no statistical testing was performed). (D) In sec‐NanoLuc conditioned medium (green, *n* = 3), the luciferase activity (RLU/s) (normalized to Milli‐Q treated sample) was abolished by Proteinase K in contrast to CD63‐NanoLuc conditioned medium (purple, *n* = 3), indicating that the systems can be used to study EV and protein secretion. A paired *t*‐test was performed with error bars representing mean ± SD. (E) ALIX and Flotillin‐1 protein expression were detected in cells and cell‐conditioned medium from wild‐type (grey, *n* = 3) and CD63‐NanoLuc (purple, *n* = 3) expressing cells. Despite a significant difference in Flotillin‐1 expression within CD63‐NanoLuc expressing cells, no difference was observed in the endogenous EV secretion to the medium. The full protein profile was visualized by Ponceau S to check for equal sample loading (Figure S6M). Two‐sample *t*‐tests were performed with error bars representing mean ± SD. Full blots are shown in Figure S6K–M. Significance levels are indicated with ns (non‐significant), *p < 0.05, **p < 0.01 and ***p < 0.001.

To increase the sensitivity, we designed a new EV reporter with CD63 instead of CD9, and we changed the reporter protein from EGFP to NanoLuc. Similar to CD9, CD63 is expressed in cultured adipocytes (Durcin et al. 2017), and fusion of NanoLuc with CD63 has been shown to improve sensitivity in vitro and in vivo (Gupta et al. 2020; Luo et al. 2020). Moreover, by fusing full‐length human CD63 to NanoLuc and the HA‐tag (CD63‐NanoLuc), we could use both specific anti‐human CD63 and anti‐HA tag antibodies for the detection of adipocyte‐derived EVs. Additionally, we created a control for general protein secretion. The secretion control (sec‐NanoLuc) was made by fusing the IgGκ signal peptide to NanoLuc and the HA‐tag. HEK293T cells transiently transfected with sec‐NanoLuc and CD63‐NanoLuc displayed EGFP fluorescence, verifying successful transfection (Figure 2A). The sec‐NanoLuc and CD63‐NanoLuc reporter proteins were readily detected in transfected cells and cell‐conditioned medium at the expected size of 20 and 60 kDa (Figure 2B), and in CD63‐NanoLuc transfected cells, human CD63 co‐localized with markers for the endoplasmic reticulum (ER), early endosome marker Rab5 and tetraspanin CD81 (Figure S2A). Size‐exclusion chromatography (SEC) of cell‐conditioned medium separated with the luciferase activity of CD63‐NanoLuc (fraction 8–10) and sec‐NanoLuc (fraction > 16), consistent with NanoLuc being associated with EVs and free protein, respectively (Figure 2C). Supporting this, the luciferase activity in sec‐NanoLuc conditioned medium was abolished by Proteinase K treatment (*p* < 0.001) (Figure 2D). CD63‐NanoLuc was not fully Proteinase K resistant (30% of the signal remained after Proteinase K treatment). However, combined with its SEC elution profiles, our results indicate that CD63‐NanoLuc is tightly EV‐associated. To assess whether overexpression of human CD63 altered endogenous EV secretion, we analyzed the protein expression of classical EV markers, that is, ALIX and Flotillin‐1, in HEK cells with or without CD63‐NanoLuc. The results indicate that human CD63 overexpression does not affect the secretion of ALIX‐ or Flotillin‐1‐positive EVs, despite a statistically significant but only slight decrease in intracellular Flotillin‐1 protein expression (*p* < 0.05) (Figure 2E).

### 3.3 Viral Delivered and Cre‐Dependent Endogenous EV‐Labelling by NanoLuc and HA Creates a Sensitive Reporter System for Tracking Circulating EVs

**FIGURE 3:**
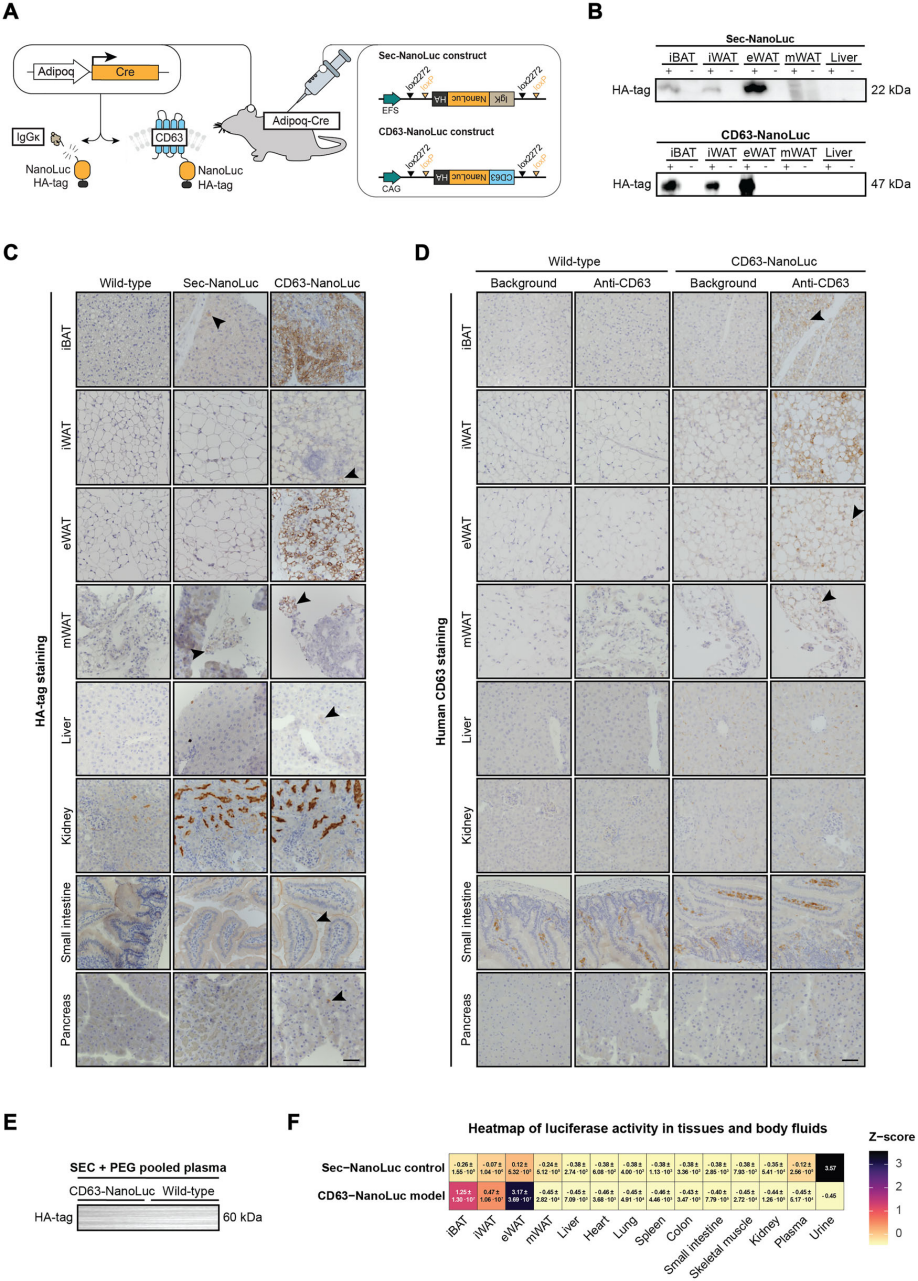
The CD63‐NanoLuc and sec‐NanoLuc mouse model. (A) The CD63‐NanoLuc and sec‐NanoLuc models were generated by AAV delivery to an adipocyte‐specific Cre‐expressing mouse line (Adipoq‐Cre). In the presence of adiponectin‐driven Cre, the double‐floxed oriented viral CD63‐NanoLuc and sec‐NanoLuc transgenes were recombined and expressed under a ubiquitous promoter (CAG or EFS), leading to the labelling of adipocyte‐derived CD63‐positive EVs or secreted protein by Nanoluciferase (NanoLuc) and hemagglutinin (HA)‐tag. (B) Adipose tissue‐specific HA‐tag protein expression was detected in both models (+) compared to wild‐type tissues (−). Full blots are shown in Figure S6N–O. (C) The HA‐tag protein expression was strongest in the adipose tissue of CD63‐NanoLuc mice compared to sec‐NanoLuc mice, while both had strong signals in the kidneys. Vague HA‐tag expression was also detected in the liver, small intestine and pancreas compared to wild‐type tissues. The scalebar represents 50 µm. (D) Human CD63 protein expression was determined between tissues with (Anti‐CD63) or without (Background) primary antibody targeting human CD63. Human CD63 protein expression was strong in adipose tissues of the CD63‐NanoLuc model. The scalebar represents 50 µm. (E) HA‐tag protein expression was not detected in size‐exclusion chromatography (SEC) purified and PEG precipitated plasma samples from chow‐fed CD63‐NanoLuc mice (*n* = 6) compared to wild‐type mice (*n* = 6). The full blot is shown in Figure S6P. (F) Heatmap of luciferase activity in tissues (*n* = 3), plasma (*n* = 3) and urine (*n* = 1) from sec‐NanoLuc and CD63‐NanoLuc mice, represented as a Z‐score ± SEM, shows the relative luciferase activity in each tissue. The CD63‐NanoLuc mice had higher luciferase activities in adipose tissues, while sec‐NanoLuc mice had the highest activities in plasma and urine. The data used to create the heatmap is shown in Figure S3D.

We designed two constructs for Cre‐dependent expression of CD63‐NanoLuc and sec‐NanoLuc in vivo by inserting the coding sequence in an inverted orientation flanked by double lox sites (Figure 3A). AAV9 delivery to Adipoq‐Cre mice would lead to irreversible adiponectin promoter‐driven Cre‐dependent expression of sec‐NanoLuc and CD63‐NanoLuc in adipocytes (Figure 3A). To assess the adipocyte‐specificity of the reporter protein expression, we designed qPCR‐based assays to determine the recombination rate of the delivered constructs. The reporter vectors were detected at high levels in ATs and liver, and to a lower extent in the heart, lung, kidneys and spleen; however, the Cre‐activated vectors, that is, recombined, were only detected in the ATs (Figure S3A), suggesting a highly selective reporter mRNA and protein expression that originates from adipocytes. Similarly, reporter protein expression was detected by western blotting for the HA‐tag in ATs but not in the liver (Figure 3B). In tissue sections, the sec‐NanoLuc control had low HA‐tag signals within ATs (Figure 3C), while the CD63‐NanoLuc showed strong HA‐tag signals in ATs (Figure 3B,C). However, kidneys from sec‐NanoLuc and CD63‐NanoLuc treated mice showed high HA antibody immunoreactivity in the kidney's proximal tubules (Figure 3C and Figure S3B). Moreover, CD63‐NanoLuc mice displayed vague HA‐antibody immunoreactive signals within the liver, brush borders of the small intestines, and pancreas (Figure 3C and Figure S3B). The EV reporter CD63‐NanoLuc uses human CD63, which can be detected selectively by antibody labelling. Similarly to the HA‐tag, human CD63 was detected in ATs, while the liver, small intestines, pancreas and kidney with immunoreactivity towards the HA‐tag, did not have detectable human CD63 signals (Figure 3D and Figure S3C). However, despite strong HA‐tag and human CD63 protein expressions within ATs, the HA‐tag was not detected in SEC‐purified and PEG‐precipitated plasma samples from six mice, further strengthening a general low sensitivity to track adipocyte‐derived EVs (Figure 3E). To assess the biodistribution of sec‐NanoLuc and CD63‐NanoLuc, we measured luciferase activity in tissue homogenates, plasma, and urine samples (Figure 3F, and individual data points in Figure S3D). Consistent with the protein levels (Figure 3B), luciferase activity was higher in ATs from CD63‐NanoLuc mice than in the sec‐NanoLuc control. In comparison, sec‐NanoLuc mice had markedly higher luciferase activity in plasma and urine (Figure 3F and Figure S3D). Thus, sec‐NanoLuc and CD63‐NanoLuc were only activated in Cre recombinase‐expressing cells, that is, adipocytes, and the biodistribution of the two reporter proteins differed, suggesting that it is possible to differentiate between ATs as EV donors and other tissues as potential EV recipients.

### 3.4 Short‐Term HFD‐Induced Obesity Shows Increased EV Abundance in eWAT iBAT and Lung

**FIGURE 4:**
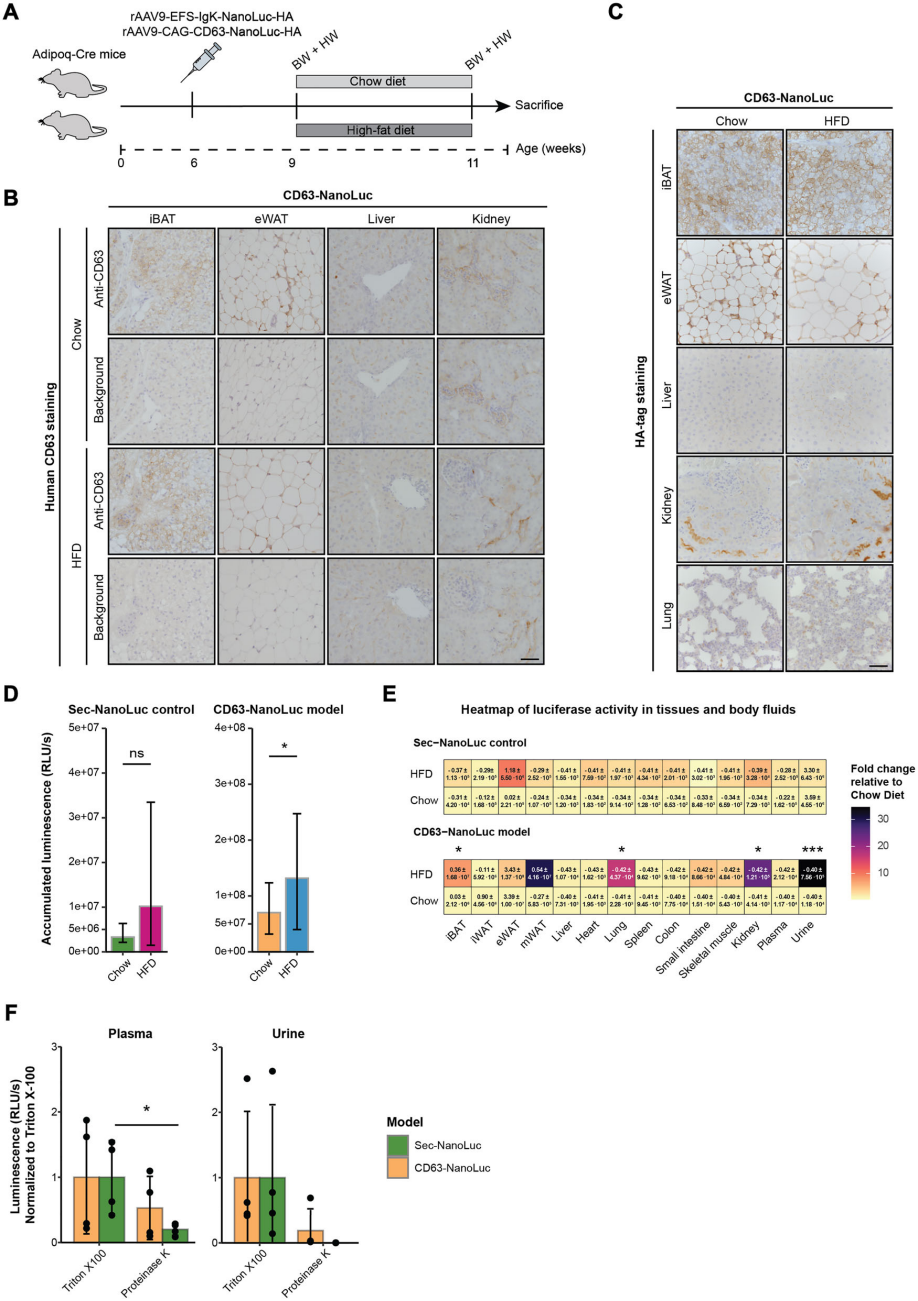
A 2‐week diet experiment using the CD63‐NanoLuc and sec‐NanoLuc models. (A) Three weeks post AAV delivery, CD63‐NanoLuc and sec‐NanoLuc mice were put on a chow diet or high‐fat diet (HFD) for 2 weeks before sacrifice. The body weight (BW) and heart weight (HW) were measured before and after the diet experiment. (B) Tissues with significantly higher luciferase activities in CD63‐NanoLuc mice on HFD than the chow diet did not have markedly higher protein expression of human CD63. The presence of human CD63 was determined between tissues with (Anti‐hCD63) or without (Background) primary antibody targeting human CD63. The scalebar represents 50 µm. (C) No difference was observed in HA‐tag protein expression between diets of CD63‐NanoLuc mice. The scalebar represents 50 µm. (D) CD63‐NanoLuc mice had significantly higher accumulated luciferase activity in HFD‐fed mice (blue, *n* = 12) compared to chow diet‐fed mice (orange, n = 13), in contrast to no significant difference between chow diet (green, *n* = 10) and HFD (pink, *n* = 10) among sec‐NanoLuc mice. Mann–Whitney tests were performed with error bars representing the median and IQR. (E) Heatmap luciferase activity in tissues and body fluids between chow diet‐ and high‐fat diet (HFD)‐fed sec‐NanoLuc and CD63‐NanoLuc mice. The Z‐score ± SEM shows the fold change in luciferase activity relative to the chow diet, which colour intensifies by an increasing Z‐score. HFD‐fed CD63‐NanoLuc mice had significantly higher luciferase activities in interscapular brown adipose tissue (iBAT), lungs, kidneys and urine. Mann–Whitney tests were performed to test for significance between diets. The data used to create the heatmap is shown in Figure S4G. (F) The luciferase activity (RLU/s) (normalized to Triton X‐100 treated sample) in plasma from sec‐NanoLuc mice (green, *n* = 4) was significantly abolished after Proteinase K treatment in contrast to plasma from CD63‐NanoLuc mice (orange, *n* = 4), suggesting that some NanoLuc signal may be encapsulated in plasma. In urine from both sec‐NanoLuc (green, *n* = 4) and CD63‐NanoLuc mice (orange, *n* = 4), the luciferase activity was completely abolished by Proteinase K, suggesting no renal filtration of EVs in CD63‐NanoLuc mice, only free proteins. A repeated measures ANOVA was performed following a pairwise *t*‐test adjustment for multiple testing using Benjamini–Hochberg correction. Error bars represent mean ± SD. Significance levels are indicated with ns (non‐significant), *p < 0.05, **p < 0.01 and ***p < 0.001.

Next, we used our CD63‐NanoLuc and sec‐NanoLuc models to study the early effects of HFD on adipocyte‐derived EV and protein abundance and biodistribution (Figure 4A). Compared to sec‐NanoLuc mice on a chow diet, we only observed a small increase in body weight of the mice on 2 weeks of HFD, but with no significant difference in the heart‐to‐body weight ratio (Figure S4A,B). For CD63‐NanoLuc mice, we observed no significant difference in body weight change or heart weight‐to‐body weight ratio between diets (Figure S4D,E). The mRNA levels of adipocyte‐specific markers (i.e., *Adipoq, Fabp4, Pparg2*) in ATs were similar between the diet groups in sec‐NanoLuc and CD63‐NanoLuc mice (Figure S4C,F). Only CD63‐NanoLuc mice had a significantly lower *Pparg2* gene expression in iWAT after 2 weeks of HFD (*p* < 0.05) (Figure S4F). Overall, the data are consistent with a phenotype in the early stages of obesity (Lee et al. 2019; Soccio et al. 2017).

Despite an apparent increase in lipid droplet size in eWAT from HFD‐fed CD63‐NanoLuc mice, we did not observe any noticeable difference in HA‐tag and human CD63 localization in ATs, liver, kidney and lungs between diets (Figure 4B,C); however, the combined NanoLuc activity for 12 different tissues, plasma, and urine samples was significantly increased by HFD, but only in CD63‐NanoLuc mice (*p* < 0.01) (Figure 4D). The higher luciferase activity in HFD‐fed CD63‐NanoLuc mice was mainly driven by significantly higher luciferase activity in iBAT (*p* < 0.05), lungs (*p* < 0.05), kidneys (*p* < 0.05) and urine (*p* < 0.001) (Figure 4E, and individual data points in Figure S4G). The higher urine luciferase activity in HFD‐fed CD63‐NanoLuc mice, combined with the presence of HA‐tag but not CD63 in kidneys, prompted us to investigate whether the NanoLuc within body fluids was encapsulated by an EV membrane. Thus, we treated CD63‐NanoLuc and sec‐NanoLuc chow plasma and urine with Proteinase K. Proteinase K treatment only significantly reduced the luciferase activity in plasma samples from sec‐NanoLuc mice (*p* < 0.05) (Figure 4F). In urine samples, on the other hand, the luciferase activity was reduced by Proteinase K treatment both in sec‐NanoLuc and CD63‐NanoLuc mice (Figure 4F), indicating that both models excrete free NanoLuc in the urine. Together, the results suggest that, although there were no notable changes in mouse phenotype, adipocyte markers or sec‐NanoLuc abundance and biodistribution, the adipocyte‐derived EV abundance and biodistribution were significantly affected after 2 weeks of HFD.

Since macrophages are potential recipient cells of EVs, we examined co‐localization of F4/80‐positive macrophages with the EV reporter HA‐tag in ATs and tissues, which had significantly higher luciferase levels in mice on HFD (Figure S5). Within ATs including iBAT and eWAT, HA‐tag signal overlapped with F4/80‐positive cells in few regions, while other areas showed no co‐localization (Figure S5A,B). In contrast, clear HA‐tag signal was detected in liver, lung and kidneys, but without apparent overlap with F4/80‐positive macrophages (Figure S5C–E).

## 4 Discussion

This study found that CD9‐EGFP labelling of adipocyte‐derived EVs did not allow monitoring of their circulating plasma levels using affinity isolation and western blotting. However, by switching to a more sensitive reporter protein—NanoLuc—and by fusion to human CD63, we created a new and sensitive EV reporter system. Compared to the truncated CD9 construct that display EGFP on the surface of EVs (Norgard et al. 2022), we used full‐length human CD63 as a fusion partner for NanoLuc. This localized NanoLuc and the HA‐tag inside EVs, protecting them from proteolytic digestion. Although the intra‐EV localization of the affinity tags limits their use for affinity isolation, human and mouse CD63 could be discriminated by the monoclonal CD63 antibody binding the extravesicular epitopes (Hildreth et al. 1991), allowing for tracking intercellular EV transport. Our EV reporter system is based on AAVs, and we could, therefore, also create a sensitive reporter for constitutive protein secretion. This control enabled us to elucidate differences in abundance and biodistribution of adipocyte‐secreted proteins and EVs in HFD‐fed mice. While our study focused on adipocytes, AAVs can target various cell types depending on the AAV serotypes (Issa et al. 2023). Moreover, new mouse models, such as the SELECTIV mouse (Zengel et al. 2023), expand AAV‐targeted tissues; thus, our sensitive protein secretion and EV reporter systems can readily be employed in health and disease models to study the biology and function of cell‐type‐specific EVs.

We used constitutive active promoters to drive reporter protein expression after Cre‐dependent recombination. While cell type‐specific promoters can be used for cell type‐specific expression of reporter proteins (Shamsi et al. 2020; Radhiyanti et al. 2021; Schroder et al. 2023), our results with the AAV vector with a shortened human adiponectin promoter to drive Cre expression in CD9‐EGFP mice highlighted a critical caveat about tissue‐specificity. When the AAVs were administered systemically, non‐adipocyte EGFP protein expression was detected in the pancreas and heart. Local injections at lower doses (Shamsi et al. 2020) can mitigate this off‐target expression, yet local AAV delivery is more invasive and limits the organs that can be targeted. AAV‐mediated delivery to cell type‐specific Cre mice, having a well‐characterized promoter to activate EV reporter protein expression, ensure a high specificity that is critical to determine, for example, cell‐to‐cell transfer of EVs.

The histological and luciferase activity analyses indicated that adipocyte‐derived EVs target distant tissues. The HA‐tag was detected in non‐adipocytes in the liver, small intestine, pancreas and kidneys. Notably, the kidney labelling with the HA‐tag is interesting because it resembles the HA‐tag labelling of the sec‐NanoLuc kidney and shows prominent labelling of the apical membrane of proximal tubular cells. These tubular cells reabsorb glomerular filtered proteins (Nielsen et al. 2016) and could imply glomerular filtration of EVs, such as EVs from the brain previously detected in urine samples (Xie et al. 2025). However, our data from the CD9‐EGFP mouse does not support glomerular filtration of plasma EVs (Norgard et al. 2022), and also human studies indicate that urine EVs are derived from the epithelial cells lining the genitourinary system (Norgard et al. 2022, Svenningsen et al. 2020, Larsen et al. 2024, Erdbrugger et al. 2021). Consistent with this, the luciferase activity in urine samples from CD63‐NanoLuc mice was not protected by a lipid membrane, indicating the urine excretion of NanoLuc was not by intact EVs. Our data, thus, imply careful analyses are crucial for establishing the distribution and cell‐targeting of EVs.

Our use of the sec‐NanoLuc revealed that an adipocyte‐specific secretion protein and EVs have different biodistributions and regulations. The sec‐NanoLuc mice had higher luciferase activity in body fluids (i.e., plasma, urine), while CD63‐NanoLuc mice had higher luciferase activities in ATs. Moreover, sec‐NanoLuc mice showed no difference in luciferase activity between chow diet and HFD. However, CD63‐NanoLuc mice on HFD had significantly higher accumulated luciferase levels, including higher levels in iBAT, lungs, kidneys and urine. Several studies show that more EVs are released during obesity, possibly due to increased mitochondrial ROS production (Auber and Svenningsen 2022; Kita et al. 2019; Han et al. 2024; Crewe et al. 2021; Norgard et al. 2023). Interestingly, the higher tissue luciferase levels were not accompanied by increased plasma adipocyte‐derived EV levels. While this could indicate a more rapid cellular uptake of adipocyte‐derived EVs in the early stages of HFD, the general circulating EV level is increased during obesity (Kwan et al. 2021), and also in mice after 2 weeks of HFD (Eguchi et al. 2016). Together, these observations suggest that the fate of cell type‐specific EVs is affected differentially during the early phases of HFD.

Consistent with our data, previous studies have shown that circulating EV levels begin to rise within 2 weeks of HFD feeding in mice (Eguchi et al. 2016). However, longer‐term interventions (8–20 weeks) are typically required to fully characterize metabolic adaptations, including fat depot expansion, redistribution and functional changes (He et al. 2020). Extending the duration would therefore provide a more comprehensive assessment of AT remodelling and EV dynamics, including their targeting to local (Liu et al. 2019; Ying et al. 2017) and distant macrophages (Zeng et al. 2022; Kang et al. 2021; Matsumoto et al. 2020). Locally within the AT, intercellular EV transfer between adipocytes and resident macrophages has been demonstrated (Liu et al. 2019; Ying et al. 2017) and, consistent with this, increased eWAT infiltration by M2 macrophages, potentially having higher phagocytotic activity than M1 macrophages (Wang et al. 2020; Jaggi et al. 2020) has been demonstrated as an acute response to HFD (Lee et al. 2011; Ji et al. 2012). Similarly, 2 weeks of HFD have been associated with increased pulmonary infiltration of macrophages (Fricke et al. 2018), which persists during long‐term HFD feeding (Namkoong et al. 2019). This may be one explanation for increased EV clearance by pulmonary macrophages during early obesity. Importantly, these changes in EV biodistribution occurred without a significant shift in body weight and adipogenic markers. Indeed, EVs derived from obese visceral AT (e.g., eWAT) have been associated with obesity's adverse effects, including insulin resistance and increased risk of cardiac dysfunction (Han et al. 2024). In contrast, BAT‐derived EVs in HFD‐fed mice decreased AT inflammation and enhanced insulin sensitivity (Han et al. 2024). The adipocyte‐derived EVs may, thus, exert essential function before the onset of obesity.

Although AT‐derived EVs have been strongly linked to obesity‐related metabolic disorders, their contribution to circulating EV levels remains debated. Some studies identify adipocytes as a major source of EV‐associated miRNAs in mice and humans (Thomou et al. 2017), supported by findings that differentiated white adipocytes secrete significantly more EVs per cell than other cell types, including brown adipocytes, hepatocytes, skeletal muscle cells and endothelial cells (Garcia‐Martin et al. 2022). These observations contrast with our previous work showing that cardiomyocyte‐ and kidney‐derived EVs can be detected in plasma and urine (Norgard et al. 2022), while adipocyte‐derived EVs remain below detection. The reason for the differences in detected adipocyte‐EV abundances between studies is unknown but highlights that changes in experimental design may affect EV biology.

Most in vivo EV biodistribution studies rely on injection of exogenously labelled EVs, which typically accumulate in liver, lungs, spleen and kidneys within the first hour (Li et al. 2022; Gupta et al. 2020; Zeng et al. 2022; Kang et al. 2021; Choi and Lee 2016) and are rapidly cleared up from circulation within 5–7 min (Auber and Svenningsen 2022; Gupta et al. 2020; Kang et al. 2021) primarily via macrophage‐dependent mechanisms (Auber and Svenningsen 2022; Zeng et al. 2022; Kang et al. 2021; Matsumoto et al. 2020; Imai et al. 2015). This highlights that immune cells, particularly macrophages, may be key regulators of EV clearance and intercellular communication. Consistent with this, we observed some co‐localization of HA‐tag signal with macrophages in iBAT and eWAT, suggesting local macrophages may act as EV scavengers. In contrast, strong HA‐tag signal in liver, lungs, and kidneys showed no apparent overlap with macrophages; yet this does not exclude their involvement, as internalized EVs including HA‐tag may undergo epitope degradation by lysosomal enzymes. However, HA‐tag signal in the liver further suggests uptake by other cell types than macrophages.

Using our EV reporter system is not without limitations. AAV‐mediated transduction of the reporter vector is associated with variable expression levels across tissues. For example, the AAV9‐mediated delivery did not transduce mWAT, limiting its applicability for studies of mWAT‐derived EVs. Moreover, variability may also arise between animals. The high sensitivity of our reporter system, however, may allow for repeated measurement of the same animal. Although this can be used to normalize for transduction levels, we minimized the effect of potential variability in transduction or expression by administering AAV prior to diet allocation and balancing experimental groups for gender, virus and diet within each litter. Moreover, the chemiluminescent assay cannot distinguish free NanoLuc from CD63‐conjugated NanoLuc, limiting conclusions about EV uptake versus clearance of free reporter protein. We used CD63 as fusion protein for NanoLuc, which may limit the sub‐types of adipocyte‐derived EVs being labelled. Still, our design of the EV reporter with extra‐ and intra‐vesicular epitopes allows both monitoring the integrity of the report construct in body fluids and the tracking of adipocyte‐derived EVs between cells.

In conclusion, we have created a sensitive NanoLuc‐based reporter system for tracking a circulating adipocyte‐secreted protein and EVs that can easily be modified to study cell‐targeting and biodistribution of cell type‐specific EVs in mice. We identified that a short‐term HFD affected the adipocyte EV biodistribution. While we used an endpoint bioluminescence assay, our EV reporter system can also be used with in vivo bioluminescence imaging to determine the adipocyte‐specific EV abundance and biodistribution dynamically during an intervention. The EV reporter system based on human CD63 could potentially enable isolation of cell type‐specific EVs using the anti‐human CD63 antibody. This could be used for demonstrating delivery of biologically relevant cargo, such as miRNAs, mRNAs or proteins, which could significantly enhance our understanding of EV biology. Overall, our AAV‐mediated NanoLuc‐based system enables monitoring cell type‐specific EV abundance and biodistribution by comparing it to a cell type‐specific secreted protein. Such data will be important on the EV secretion and clearance dynamics are crucial for unravelling EV abundance and biodistribution during health and disease and potentially developing targeted therapies to modulate EV‐mediated intercellular communication.

## Author Contributions

**Didde Riisager Hansen**: conceptualization, methodology, data curation, investigation, validation, formal analysis, funding acquisition, visualization, project administration, resources, writing – original draft. **Rugivan Sabaratnam**: investigation, writing – review and editing, methodology, validation. **Lasse Bach Steffensen**: writing – review and editing, visualization, methodology, funding acquisition, resources, data curation, formal analysis. **Per Svenningsen**: conceptualization, investigation, writing – original draft, validation, funding acquisition, visualization, project administration, supervision, resources, methodology, writing – review and editing, formal analysis, data curation.

## Conflicts of Interest

The authors declare no conflicts of interest.

## Supporting information




**Supporting Information**: jev270243‐sup‐0001‐FigureS1.png

**Supporting Information**: jev270243‐sup‐0002‐FigureS2.png

**Supporting Information**: jev270243‐sup‐0003‐FigureS3.png

**Supporting Information**: jev270243‐sup‐0004‐FigureS4.png

**Supporting Information**: jev270243‐sup‐0005‐FigureS5.png

**Supporting Information**: jev270243‐sup‐0006‐FigureS6.png

**Supporting Information**: jev270243‐sup‐0007‐SuppMat.docx

## Data Availability

The data that support the findings of this study are available from the corresponding author upon reasonable request.
