# Supplementary figures and images for: A Sensitive Reporter Mouse Model to Study Adipocyte‐Derived Extracellular Vesicles In Vivo
Source: J Extracell Vesicles. 2026 Feb 19;15(2):e70243. doi: 10.1002/jev2.70243 (PMC12919364; doi:10.1002/jev2.70243)

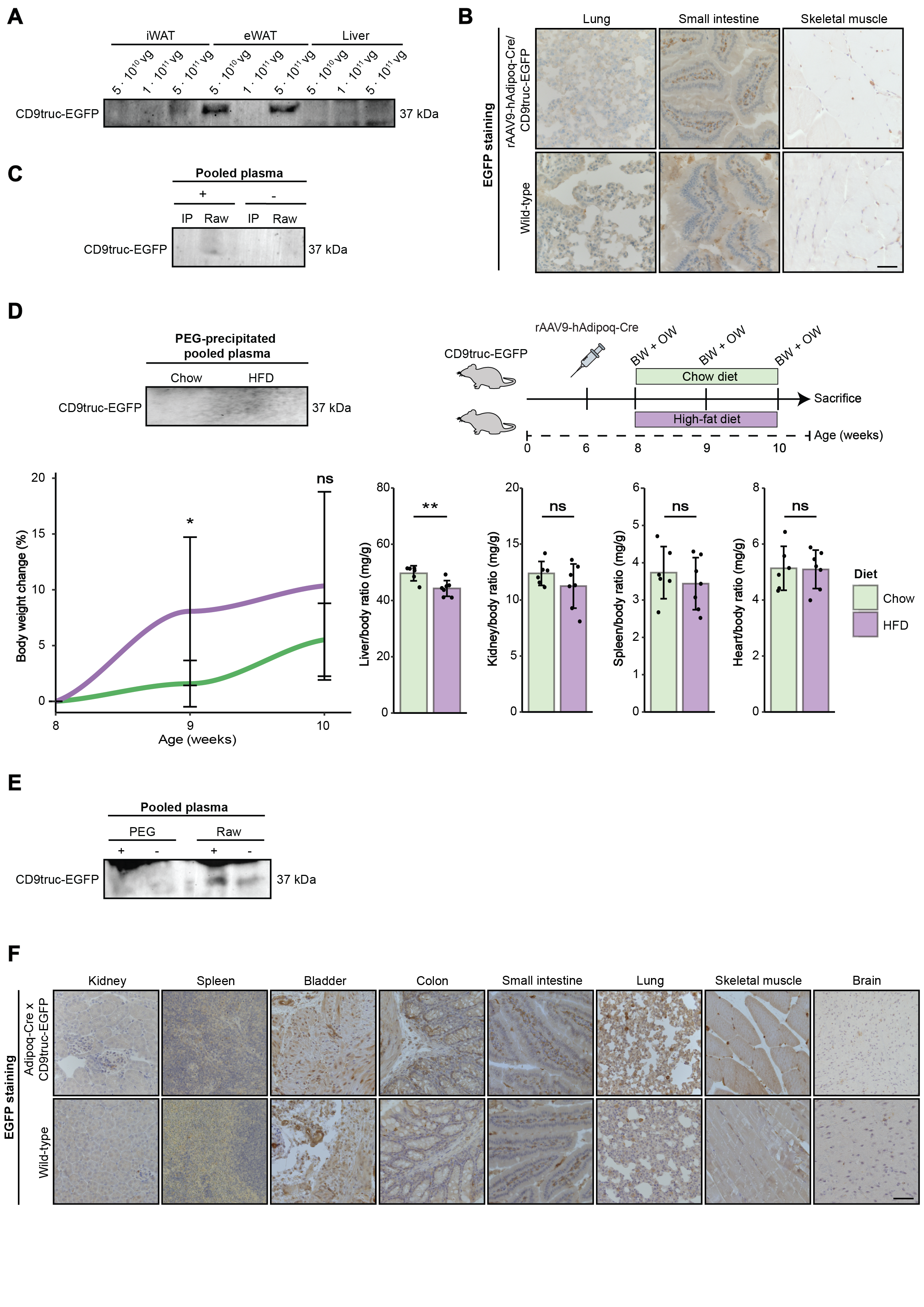

Supplement: Supplementary file 1 — Supporting Information: jev270243‐sup‐0001‐FigureS1.png [file JEV2-15-e70243-s006.png]

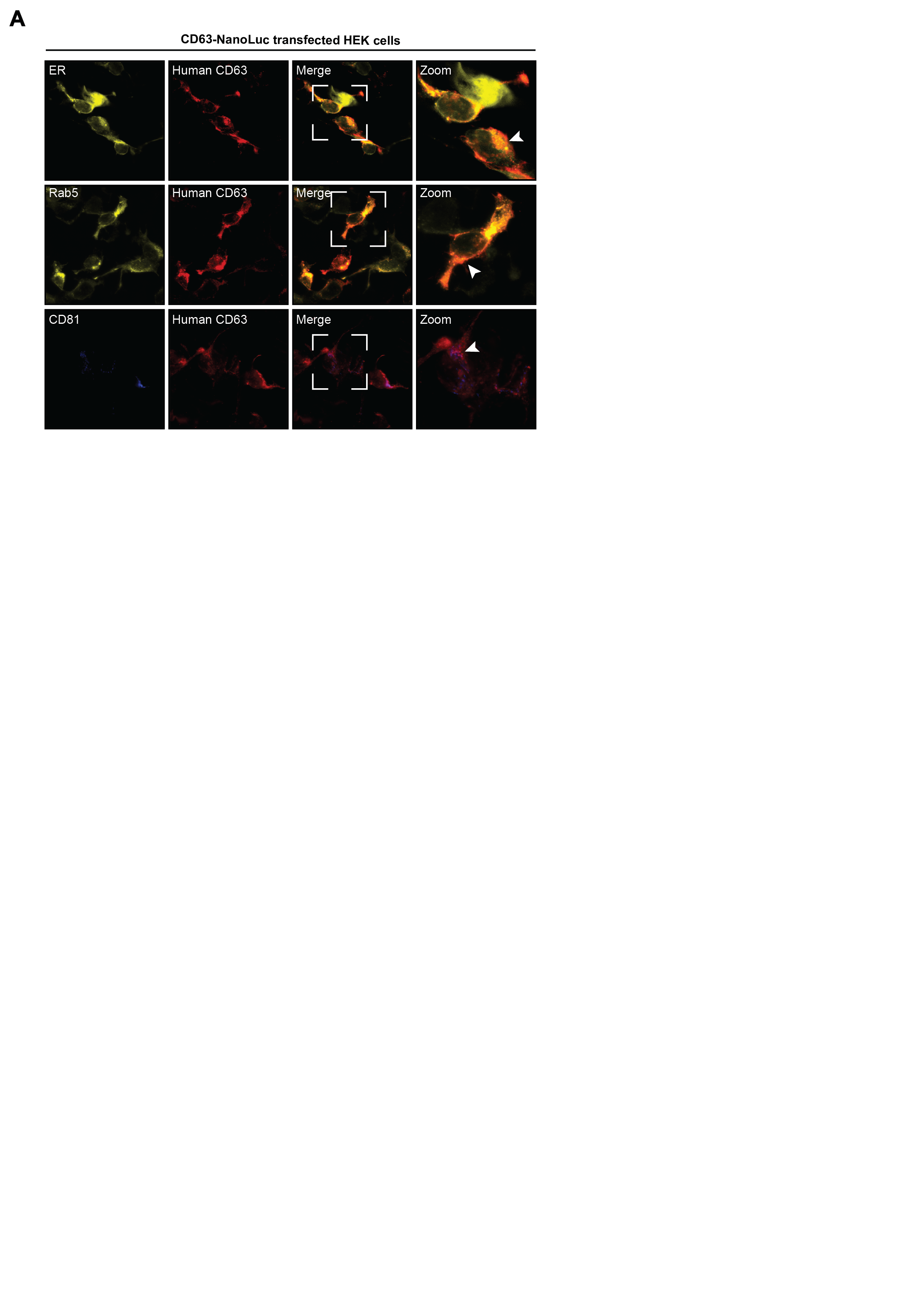

Supplement: Supplementary file 2 — Supporting Information: jev270243‐sup‐0002‐FigureS2.png [file JEV2-15-e70243-s005.png]

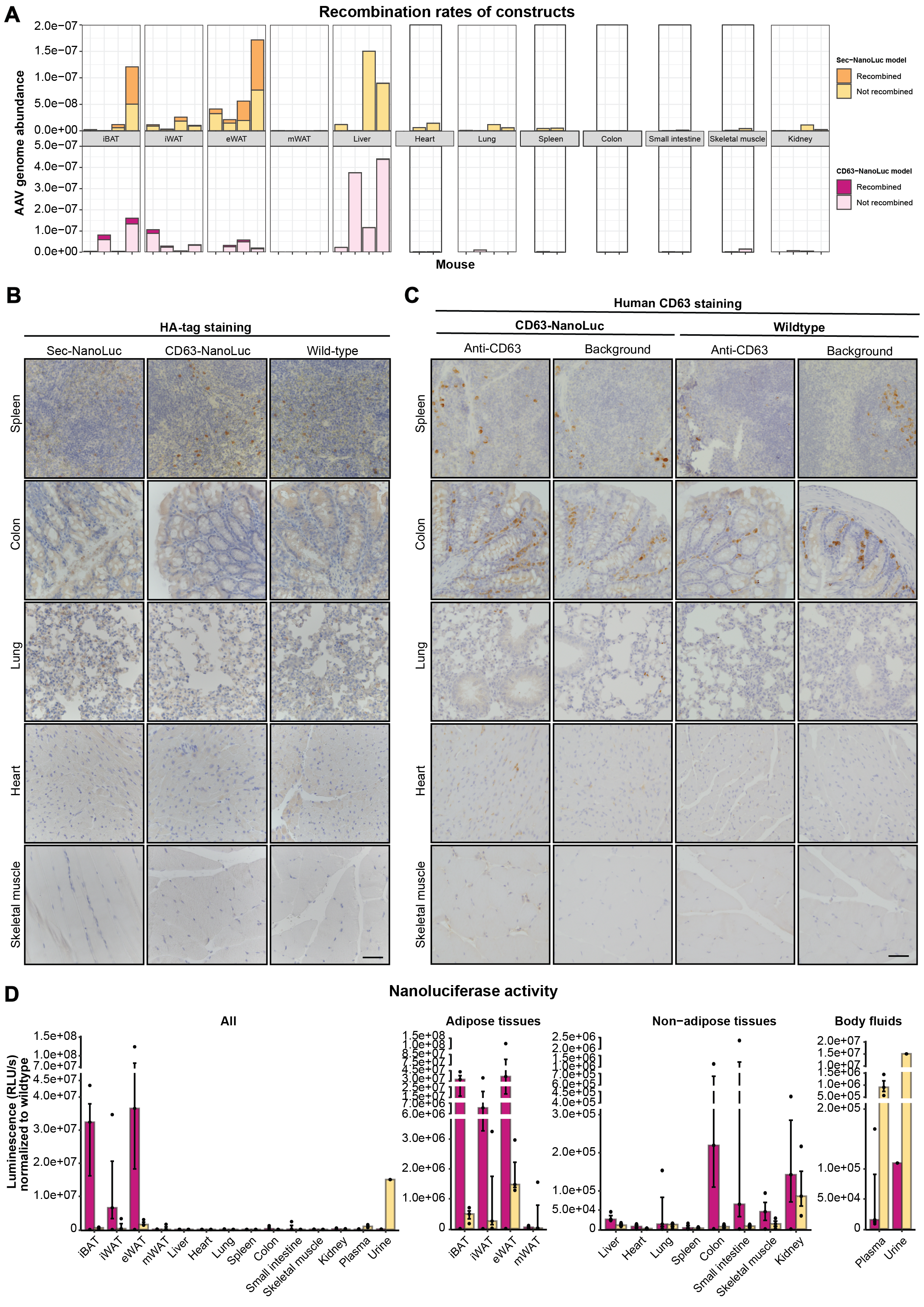

Supplement: Supplementary file 3 — Supporting Information: jev270243‐sup‐0003‐FigureS3.png [file JEV2-15-e70243-s004.png]

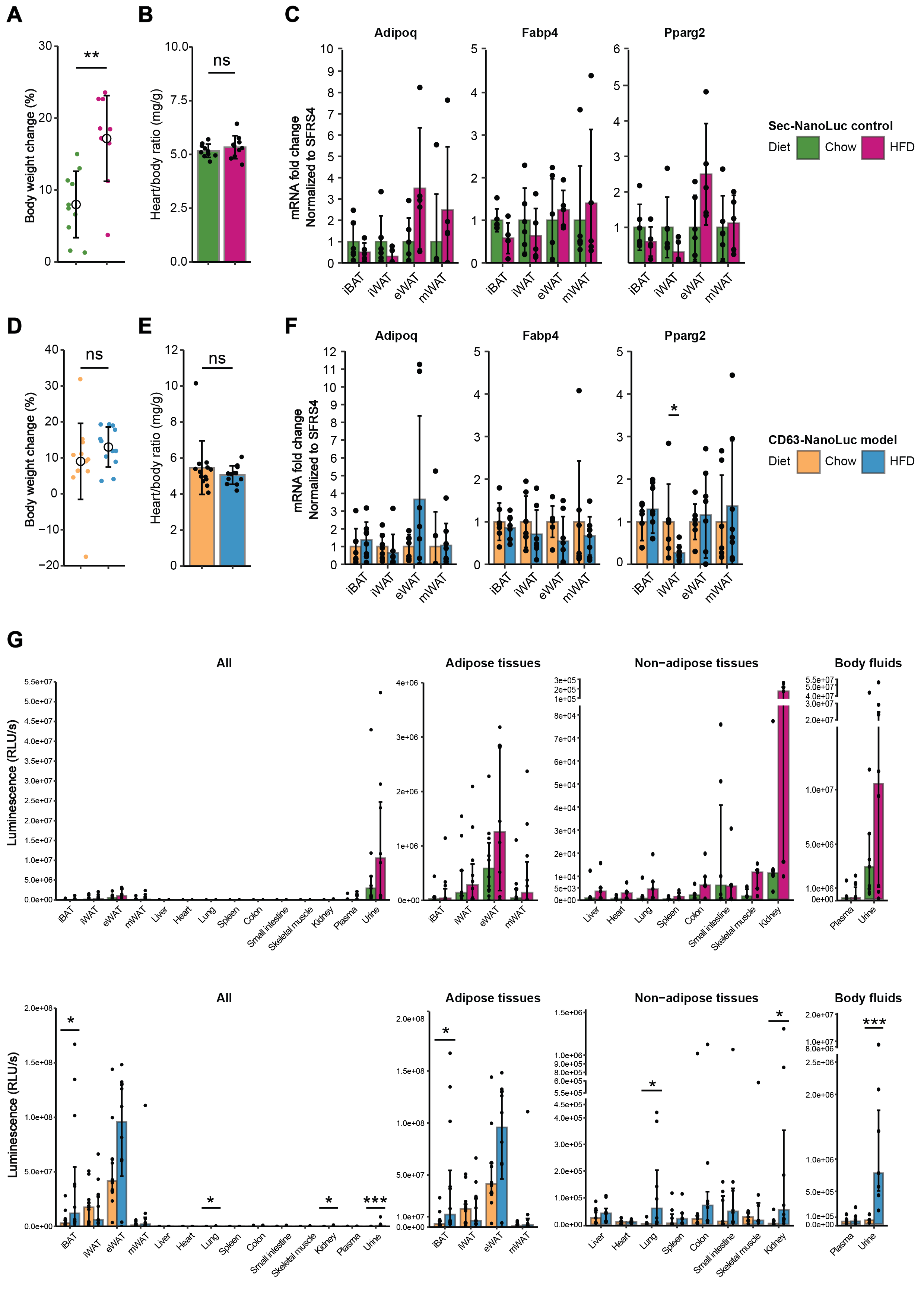

Supplement: Supplementary file 4 — Supporting Information: jev270243‐sup‐0004‐FigureS4.png [file JEV2-15-e70243-s007.png]

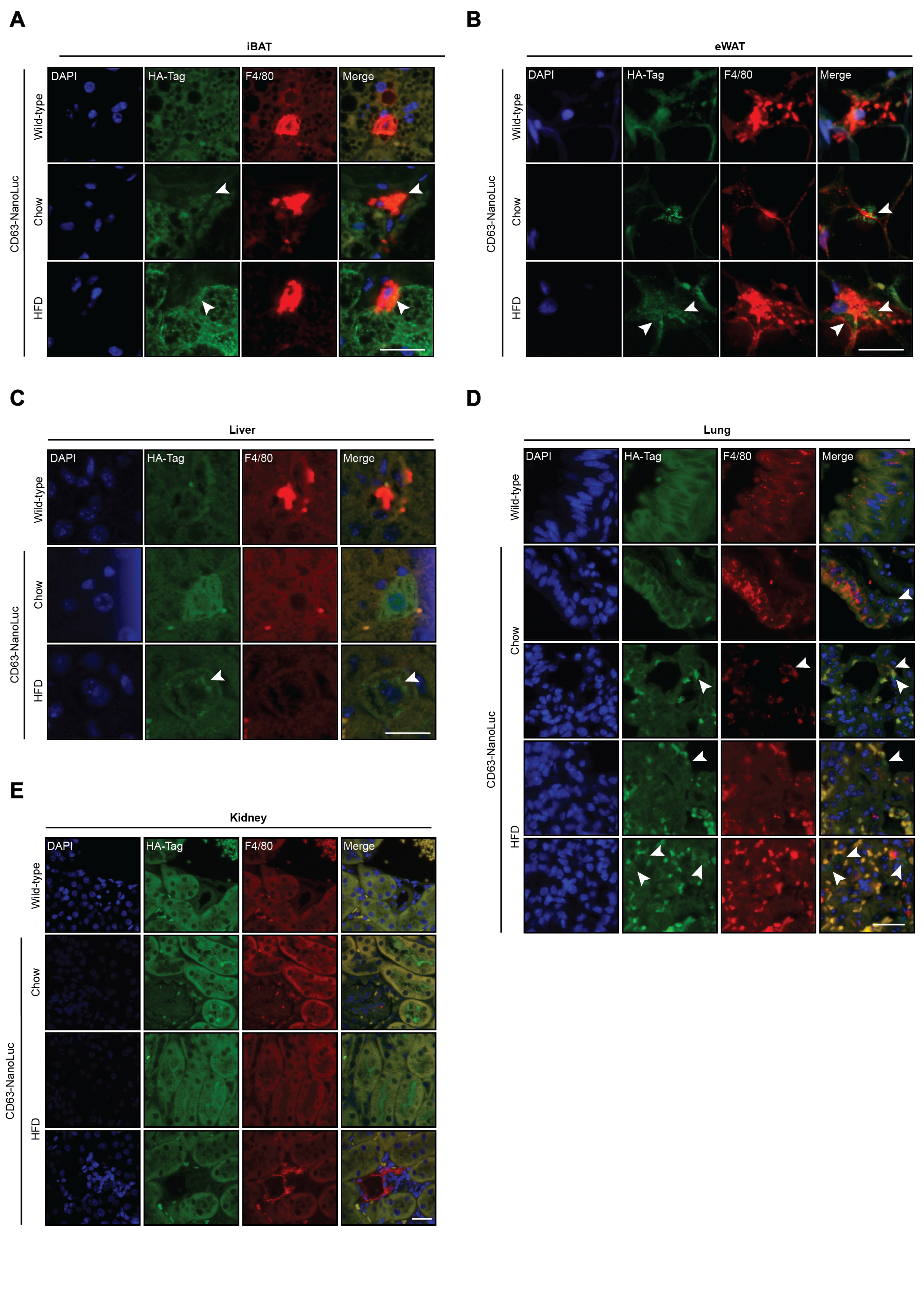

Supplement: Supplementary file 5 — Supporting Information: jev270243‐sup‐0005‐FigureS5.png [file JEV2-15-e70243-s002.png]

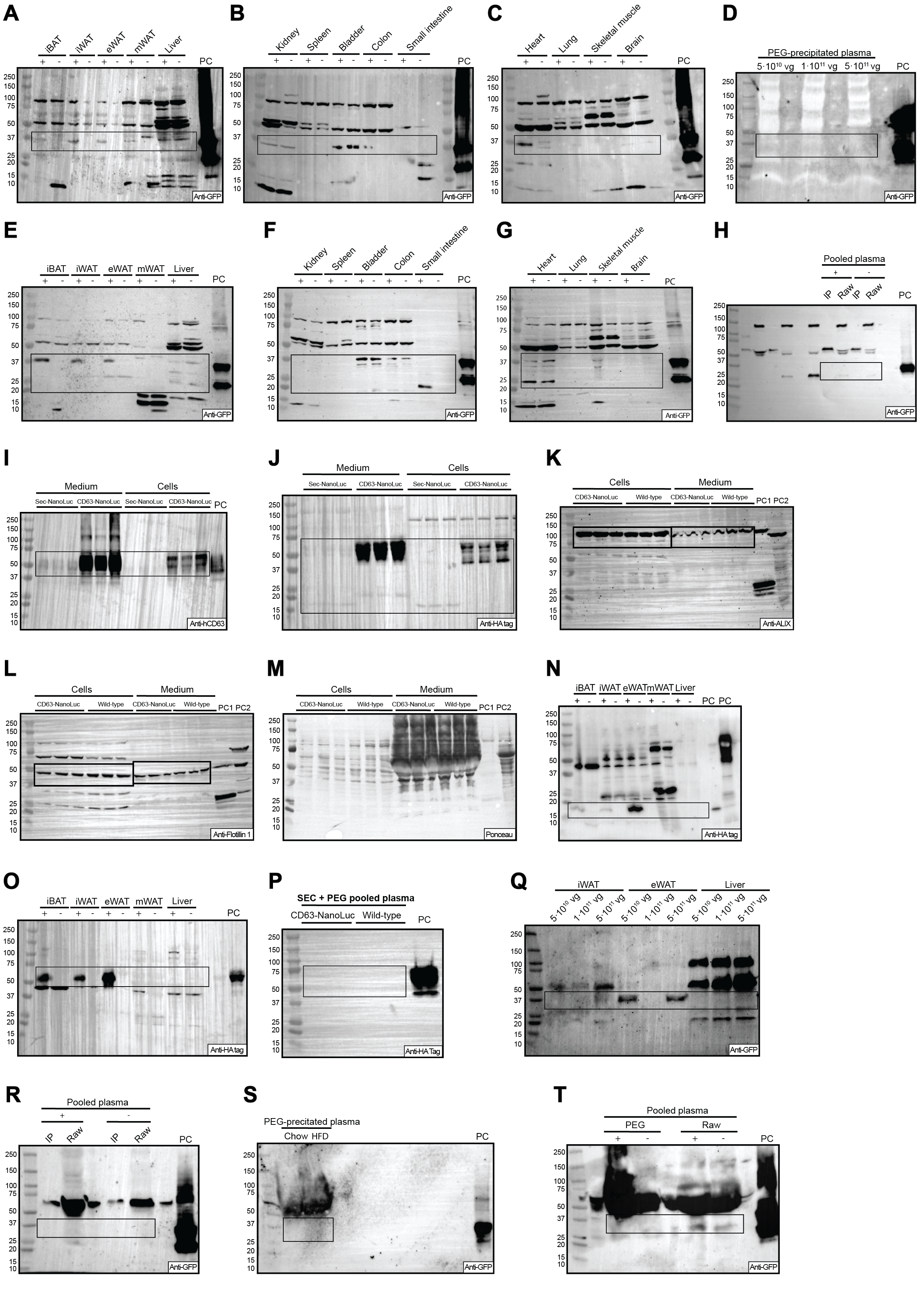

Supplement: Supplementary file 6 — Supporting Information: jev270243‐sup‐0006‐FigureS6.png [file JEV2-15-e70243-s003.png]
