# Supplementary material for: A Sensitive Reporter Mouse Model to Study Adipocyte‐Derived Extracellular Vesicles In Vivo
Source: J Extracell Vesicles. 2026 Feb 19;15(2):e70243. doi: 10.1002/jev2.70243 (PMC12919364; doi:10.1002/jev2.70243)
Supplement: Supplementary file 7 — Supporting Information: jev270243‐sup‐0007‐SuppMat.docx [file JEV2-15-e70243-s001.docx]

# Supplementary figure captions

**Figure S1:** **Additional data using the CD9-EGFP reporter system by AAV-mediated and transgenic mouse breeding to obtain adipocyte-specific Cre activation. A)** To test the AAV dose efficiency, CD9truc-EGFP protein expression was evaluated in tissues from mice receiving different doses of rAAV9-hAdipoq-Cre, of which a dose of 5 · 10^11^ viral genomes (vg) was used. The full blot is shown in Fig. S6Q. **B)** Staining of EGFP showed no off-target effects in the small intestine, lungs, and skeletal muscle in rAAV9-hAdipoq-Cre/CD9truc-EGFP mice. The scalebar represents 50 µm. **C)** No CD9truc-EGFP was detected in immunoprecipitated (IP) pooled plasma from chow-fed mice (n=7) receiving 5 · 10^11^ vg rAAV9-hAdipoq-Cre (+) compared to wild-type (-). The full blot is shown in Fig. S6R. **D)** CD9truc-EGFP protein expression was not detected in PEG-precipitated plasma samples from chow diet- (n=3) and high-fat diet (HFD)-fed (n=3) CD9truc-EGFP mice receiving 5 · 10^11^ vg of rAAV9-hAdipoq-Cre. The full blot is shown in Fig. S6S. Mice were put on a chow diet (green, n=6) or HFD (purple, n=7) for 2 weeks, where body weight (BW) and organ weight (OW), of the liver, kidneys, spleen and heart weight were measured at the start, mid and end of the experiment. The body weight change (%) did not significantly differ between diet groups, while the relative liver weight to body weight (mg/g) was significantly lower in the HFD group. Two-sample t-tests were performed with error bars representing mean ± SD. Significance levels are indicated with ns (non-significant), * (p<0.05), ** (p<0.01), and *** (p<0.001). **E)** In chow-fed mice receiving a higher dose of 1 · 10^12^ vg of rAAV9-hAdipoq-Cre (+), no CD9truc-EGFP detected in PEG-precipitated pooled plasma (n=2) compared to wild-type plasma (-). The full blot is shown in Fig. S6T. **F)** EGFP staining showed no present CD9truc-EGFP expression in the kidney, spleen, bladder, colon, small intestine, lung, skeletal muscle, and brain tissue of Adipoq-Cre x CD9truc-EGFP mice. The scalebar represents 50 µm.

**Figure S2:** **Fluorescent evaluation of co-localization of human CD63 with endoplasmic reticulum, Rab5 and CD81 in vitro. A)** Immunocytochemistry data of HEK cells expressing CD63-NanoLuc (red) co-transfected with mCherry-ER (yellow), mRFP-Rab5 (yellow), or mTagBFP2 (blue). Data shows some co-localization of human CD63 with the endoplasmic reticulum (ER), the early endosome marker Rab5, and tetraspanins CD81.

**Figure S3:** **Data from CD63-NanoLuc and sec-NanoLuc mouse models. A)** The AAV genome abundance measured as the mean relative expression (2^-Ct^) of Cre-recombined versus the mean relative expression (2^-Ct^) of the non-recombined construct in tissues from CD63-NanoLuc (n=2-4) and sec-NanoLuc (n=2-4) mice normalized to the mean relative expression (2^-Ct^) of wild-type tissues (n=4-5). Data shows adipocyte-specific Cre-induced activation of constructs enabling differentiation between EV donors and EV recipients. **B)** HA-tag protein expression was not detected in the spleen, colon, lungs, heart and skeletal muscle from CD63-NanoLuc and sec-NanoLuc mice compared to wild-type mice. The scalebar represents 50 µm. **C)** Additionally, no human CD63 expression was detected in the spleen, colon, lungs, heart and skeletal muscle from CD63-NanoLuc mice compared to wild-type mice. The presence of human CD63 was evaluated between tissues with (Anti-hCD63) or without (Background) primary antibody. The scalebar represents 50 µm. **D)** The luciferase activity (RLU/s) in tissues (n=3), plasma (n=3), and urine (n=1) from the CD63-NanoLuc and sec-NanoLuc mice was overall significantly higher than baseline wild-type luciferase activities. The CD63-NanoLuc mice had higher activities in adipose tissues (iBAT, iWAT, eWAT) compared to sec-NanoLuc mice, which, in contrast, had higher plasma and urine activities. Error bars represent the median and IQR (no statistical tests were performed).

**Figure S4:** **A two-week diet experiment using CD63-NanoLuc and sec-NanoLuc mice.** For sec-NanoLuc mice on chow diet (green, n=10) or HFD (pink, n=10), **A)** a significant increase in body weight change (%) was observed in HFD-fed mice, with **B)** no significant differences in relative heart weight to body weight ratio (mg/g) or in **C)** the fold change in mRNA expression of Adipoq, Fabp4 and Pparg2 (normalized to SFRS4) in adipose tissues between diets. Two-sample t-tests were performed with error bars representing mean ± SD. For CD63-NanoLuc mice on the chow diet (orange, n= 13) or HFD (blue, n= 12), no significant difference was observed in **D)** body weight change (%) or in **E)** relative heart to body weight ratio (mg/g), and in **F)** the mRNA fold change in Adipoq, Fabp4, and Pparg2 (normalized to SFRS4), a significant decrease in Pparg2 expression in iWAT was detected in HFD-fed mice. Two-sample t-tests were performed with error bars representing mean ± SD. **G)** The luciferase activity (RLU/s) between chow diet (green, n=10) and high-fat diet (HFD) (pink, n=10) in tissues from sec-NanoLuc mice showed no significant significance, while HFD-fed CD63-NanoLuc mice (blue, n=12) had significantly higher activities in interscapular brown adipose tissue (iBAT), lungs, kidneys and urine than the chow diet group (orange, n=13). Mann-Whitney tests were performed with error bars representing the median and IQR. Significance levels are indicated with ns (non-significant), * (p<0.05), ** (p<0.01), and *** (p<0.001).

**Figure S5:** **Immunofluorescent evaluation of co-localization of HA-Tag and macrophage marker F4/80 in tissues of chow diet and HFD-fed CD63-NanoLuc mice**. Overlapping HA-tag (green) and F4/80 (red) was observed in adipose tissues including **A)** interscapular brown adipose tissue (iBAT) and **B)** epididymal white adipose tissue (eWAT) compared to wild-type mice. However, no clear evidence of co-localization was observed in **C)** liver, **D)** lungs and **E)** kidneys, but HA-tag signal was observed in non-macrophagic (F4/80+) cell types within the liver. No clear difference was between diets. The scalebars represents 50 µm.

**Figure S6:** **Full-length western blots.** Cropped areas used for figures are highlighted. A-H) Blots used for Fig. 1. I-M) Blots used for Fig. 2 (Fig. S6M shows full protein profile used as loading control for Fig. S6K-L). N-P) Blots used for Fig. 3. Q-T) Blots used for Fig. S1. The positive control (PC) for EGFP shows CD9truc-EGFP and free EGFP at 37 kDa and 29 kDa, respectively. The positive control (PC) for human CD63 shows CD63 at 60 kDa. The positive controls (PC) for HA-tag shows HA-tag at 60 kDa and 25 kDa for the CD63-NanoLuc and sec-NanoLuc model, respectively. The positive controls for ALIX show ALIX at around 95-100 kDa. The positive controls (PC) for Flotillin-1 shows Flotillin-1 at 48 kDa.
